# Membrane Permeabilities of Ascorbic Acid and Ascorbate

**DOI:** 10.3390/biom8030073

**Published:** 2018-08-17

**Authors:** Christof Hannesschlaeger, Peter Pohl

**Affiliations:** Institute of Biophysics, Johannes Kepler University Linz, Gruberstr. 40, 4020 Linz, Austria; Christof.Hannesschlaeger@jku.at

**Keywords:** scorbic acid, ascorbate, passive membrane permeability, vitamin C, membrane permeation, weak acid permeation

## Abstract

Vitamin C (VC)—a collective term for the different oxidation and protonation forms of ascorbic acid (AscH)—is an essential micronutrient that serves as (i) a potent antioxidant and (ii) a cofactor of a manifold of enzymatic processes. Its role in health is related to redox balance maintenance, which is altered in diseases such as obesity, cancer, neurodegenerative diseases, hypertension, and autoimmune diseases. Despite its importance, VC uptake has been poorly investigated. Available literature values for the passive membrane permeability *P* of lipid bilayers for AscH scatter by about 10 orders of magnitude. Here, we show by voltage clamp that $P^{-}$ of AscH’s anionic form (ascorbate ${\mathrm{Asc}}^{-}$) is negligible. To cross the membrane, ${\mathrm{Asc}}^{-}$ picks up a proton in the membrane vicinity and releases it on the other side of the membrane. This leads to a near-membrane pH drop that was visualized by scanning pH microelectrodes. The AscH concentration dependent pH profiles indicated $P\;=\;1.1\;\pm \;0.1\;\times \;10^{-8}\;\mathrm{cm}/s$. Thus, AscH’s *P* is comparable to that of sorbitol and much lower than that of other weak acids like acetic acid or salicylic acid. The observation suggests that the capacity of the passive transcellular transport pathway across the lipid matrix does not suffice to ensure the required VC intake from the gastrointestinal tract.

## 1. Introduction

Vitamin C (VC) encompasses several vitamers that differ in their protonation and oxidation states and include ascorbic acid (AscH). Vitamin C deficiency results in scurvy [1]. Membrane proteins like the sodium-ascorbate cotransporters SVCT1 and SVCT2 transport reduced ascorbate, thereby contributing to VC homeostasis in the human body [2]. However, SVCT1 knockout only marginally affected intestinal VC adsorption in mice [3], suggesting a role for redundant glucose transporters (GLUT transporters) or significant spontaneous membrane permeability *P* of AscH that may ensure sufficient intestinal VC uptake. A more quantitative investigation of SVCT’s biological significance requires knowledge of AscH’s passive membrane permeability. Yet, surprisingly little is known. Since AscH is a weak acid with one of its pK values being equal to 4.17 [4], it may be anticipated to passively permeate biological membranes—similarly to other weak acids such as salicylic acid [5] or acetic acid [6,7]. The second proton release reaction with a pK value of 11.57 [8] yields a bivalent anion that due to (i) its extremely low concentration at physiological pH values and (ii) the increased desolvation (Born) energy for bivalent ions cannot make a significant contribution to passive transmembrane VC flux.

With $P\;=\;10^{-10}\;\mathrm{cm}/s$ [9] the only available literature value differs by up to 10 orders of magnitude from values that maybe derived from (i) the membrane permeability $P^{-}$ to the ascorbate anion (${\mathrm{Asc}}^{-}$) [10] or (ii) the oil water partition coefficient of AscH [11]. The goal of the present work was the characterization of AscH passive membrane transport processes. We did so by exploiting scanning electrochemical microscopy in the vicinity of planar lipid bilayers, as this method previously yielded robust values for various acids and bases [5,6,12,13].

Membrane conductivity measurements rendered an upper estimate for $P^{-}$, which excludes ${\mathrm{Asc}}^{-}$ from making a significant contribution to the passive membrane permeation of VC.

## 2. Materials and Methods

### 2.1. Planar Lipid Bilayer

Free standing planar lipid bilayers from *Escherichia coli* polar lipid extract (PLE; Avanti Polar Lipids, Alabaster, AL, USA) dissolved in *n*-decane (10 mg/mL) were spread over an aperture (~400 µm in diameter) in a 25 μm thick Teflon septum which was pretreated with 1 μL of the lipid solution [14]. At each side of the bilayer, an Ag/AgCl electrode was placed which allowed the application of (i) a triangle AC-voltage (320 Hz, 10 mV peak-to-peak) via an npi-VA-10x current amplifier (npi Elektronik, Tamm, Germany) and (ii) an additional DC bias which is necessary to observe membrane formation. 

The membrane conductivity was calculated via Ohm’s law. An A/D converter controlled by the WinWCP 4.0.6 software (University of Strathclyde, Glasgow, UK) digitized the analog output of the current amplifier equipped with a 5 GΩ headstage at a sampling rate of 0.116 s. The ampilifier’s 4-pole Bessel low pass filter was set to 50 Hz. All measurements were performed at room temperature (22 °C).

### 2.2. Ascorbic Acid

Ascorbic Acid spontaneously oxidizes at physiological pH and ambient oxygen concentrations, producing ascorbyl radical and dehydroascorbic acid. This process can be accelerated by light, heat, increasing pH, and the presence of contaminating free iron or copper [15]. To prevent oxidation, we (i) prepared the aqueous AscH containing solutions freshly each day with degassed ultra-pure (Milli-Q, Merck Millipore, Burlington, MA, USA; hence metal-free) water at a pH of 4.25. We were forced to use high millimolar (i.e., physiologically irrelevant) concentrations of ascorbate in our experiments in order to detect transport. Yet the observation of concentration independent *P* values (see below) indicated the absence of non-standard chemical reactions.

### 2.3. Scanning Electrochemical Microscopy

The setup and measurement procedure for scanning electrochemical microscopy are described elsewhere in detail [16]. Briefly, a borosolicate glass capillary is pulled to yield a tip diameter of about 2 μm. After the tip is bent to a right angle, it is silanized inside with bis(dimethylamino)dimethylsilane to create a hydrophobic surface. A protonophore cocktail (Hydrogen ionophore cocktail II, Fluka) filled into the very tip of the pipette renders the electrode pH sensitive. The pipette is filled with 500 mM KCl solution, and a thin Ag/AgCl wire is inserted. This microelectrode is mounted onto a hydraulic microdrive manipulator (Narishige, Tokyo, Japan), which allows scanning of pH profiles perpendicular to the bilayer surface. The microelectrode and a reference electrode are located in the same compartment of the measurement chamber. Both are connected to a Keithley 6514 electrometer (Keithley Instruments, Cleveland, OH, USA) that is controlled via an IEEE interface and a custom-written LabView program (National Instruments, Austin, TX, USA). The solutions in the chamber are agitated with magnetic stirrer bars to diminish the unstirred layers (USL) in the vicinity of the membrane. Prior to every experiment, the pH-sensitivity of the microelectrode is calibrated.

**Figure 1:**
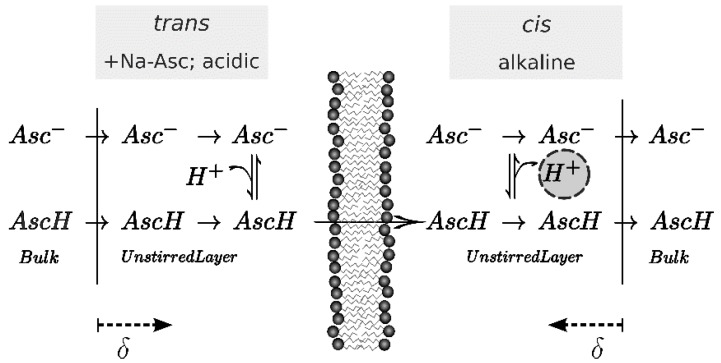
Scheme of the effect of pH shift upon one-sided addition of Na-Asc to the trans side of the membrane where an acidic pH ensures a high ascorbic acid (AscH) concentration. Once arriving on the alkaline cis-side, almost all acid molecules deprotonate and release a proton (encircled). Hence, the pH drops on the cis in the vicinity of the membrane. Since each compartment is stirred, this effect is occurring only within the unstirred layer of thickness *δ*. Scanning electrochemical microscopy serves to measure this pH shift.

The buffer for the scanning electrochemical microscopy measurement consists of 100 mM KCl, 0.5 mM β-alanine, 0.3 mM KH_2_PO_4_ adjusted to pH 4.2. All chemicals were purchased from Sigma-Aldrich (Vienna, Austria). All solutions are sterile filtered. Ascorbic acid gradients are built up adding to the *trans*-side aliquots from a 2 M sodium-l-ascorbate solution (pH 4.2) that is prepared in measurement buffer. At the beginning of the measurement pH on both sides of the membrane is 4.2. Subsequently, pH on the microelectrode side (*cis*-side) is augmented to 6.5 by KOH addition. The pH gradient acts to decrease the AscH on the *cis*-side. In turn, the transmembrane AscH flux increases, thereby giving raise to resolvable pH changes within the *cis* USL (compare Figure 1). After an incubation time of roughly 15 min, the microelectrode is stepwise moved towards the membrane with a velocity of 4 μm/s. The position of the membrane is judged from a saltatory increase in microelectrode voltage [17]. Profiles are averaged over at least two scans.

### 2.4. Calculation of P^−^ from Membrane Conductivity Measurements

An electrochemical gradient of an ion *s* of valency *z_s_* with membrane permeability *P_s_* across a planar lipid bilayer causes a current of density *j_s_*. In this study, symmetrical buffer conditions are chosen. The Goldman-Hodgkin-Katz (GHK) flux equation [18,19,20] connects these quantities. We calculate *P_s_* from the specific membrane conductivity induced by the ion *s* ($g_{s}=j_{s}/U$) by using the GHK flux equation (Equation (1)) for symmetrical conditions.

(1) $$\;P_{s}=\frac{R\cdot T}{z_{s}{}^{2}\cdot F^{2}}\cdot \frac{g_{s}}{\left[s\right]}\;$$

Conductivity measurements were performed for different Na-Asc concentrations at various pH values since around its pK, some weak acids tend to form dimers of the acid and the conjugated base molecule [21]. Because of its charge, the dimer has a presumably lower membrane permeability than the acid form, but a substantially larger membrane permeability than the conjugated base due to its increased volume which lowers the self-solvation energy for entering the lipid environment [5].

The buffer for the conductivity measurement consists of 100 mM NaCl, 10 mM HEPES, pH 7.5. The volume in the measurement chamber is partially replaced with a solution of 1 M NaAsc, 100 mM NaCl, 10 mM HEPES pH 7.5. Additions of HCl lower the pH.

### 2.5. Calculation of Permeability from pH Profiles in the Unstirred Layers

Shifts in the local pH adjacent to the membrane are indicative of *P* [6]. In addition to *P* they are governed by chemical reactions with buffer molecules and the diffusivity *D_i_* of all reactants. Consequently, calculation of *P* should take into account both buffer and acid concentrations. Accordingly, we numerically solved a system of differential equations for both sides of the membrane that describes the diffusion (Equation (2)) and expenditure in chemical reactions (Equation (3)). Membrane permeation is assumed to be so slow that all chemical reactions are in equilibrium. The equilibrium constants ${(K}_{i})$ of protons $\left(H^{+}\right)$ and hydroxide anions $\left({\mathrm{OH}}^{-}\right)$ (Equation (4)), ascorbic acid AscH, and ascorbate ${\mathrm{Asc}}^{-}$ (Equation (7)), as well as the protonated and deprotonated forms of the zwitterionic β-alanine ${\mathrm{BH}}^{+}$ and B (Equation (5)), and the protonated and deprotonated forms of the phosphate buffer at its second pK, ${\mathrm{HPO}}_{4}^{2-}$ and $H_{2}{\mathrm{PO}}_{4}^{-}$ (Equation (6)) are listed in Table 1 along with the diffusivities of the different species (denoted by index *i* = 1 … 8) that participate in protonation reactions. The magnitude and unit of the equilibrium constants K are calculated from the respective pK value and type of reaction they describe.

(2) $$\;J_{i}\left(x\right)=-D_{i}\cdot \frac{d\;c_{i}\left(x\right)}{d\;x},\;$$

(3) $$\;\frac{d\;J_{i}\left(x\right)}{d\;x}=R_{i}\left(c_{i=1\ldots 8}\left(x\right)\right),\;$$

(4) $$\;H^{+}+{\mathrm{OH}}^{-}⇋H_{2}O\;K_{1,2}=c_{1}\left(x\right)\cdot c_{2}\left(x\right),\;$$

(5) $$\;H^{+}+B⇋{\mathrm{BH}}^{+}\;K_{3,4}=\frac{c_{1}\left(x\right)\cdot c_{3}\left(x\right)}{c_{4}\left(x\right)},\;$$

(6) $$\;H^{+}+{\mathrm{HPO}}_{4}^{2-}⇋H_{2}{\mathrm{PO}}_{4}^{-}\;K_{5,6}=\frac{c_{1}\left(x\right)\cdot c_{5}\left(x\right)}{c_{6}\left(x\right)},\;$$

(7) $$\;H^{+}+{\mathrm{Asc}}^{-}⇋\mathrm{AscH}\;K_{7,8}=\frac{c_{1}\left(x\right)\cdot c_{7}\left(x\right)}{c_{8}\left(x\right)}.\;$$

Phosphate has three pK values: 2.12, 7.21 and 12.67 [22]. Its buffering capacity is only significant on the cis-side since pK = 7.21 is close to pH ~6.5. The trans side is mainly buffered by β-alanine (pK close to pH). Thus, the low buffering capacity of the phosphate buffer (pK far from pH) can be ignored there. All membrane impermeable substances obey a no-flux-condition at the water-membrane interfaces (*x* = 0 in nomenclature of the figures) (Equation (8)), except for AscH which flows along its transmembrane gradient, $∆c_{8}$ (Equation (9)).

(8) $$\;J_{i\neq 8}\left(0\right)=0\;$$

(9) $$\;J_{8}\left(0\right)=-P\cdot ∆c_{8}\;$$

The size δ of the unstirred layer is defined in terms of the concentration gradient at the membrane water interface (*x* = 0, Equation (10)).

(10) $$\;\delta =\frac{dc_{1}}{dx}{|}_{x\;=\;0}\;$$

A linear fit to the profile in the first 100 μm adjacent to the membrane serves to determine *δ*. Subsequently, we used Mathematica 9 (Wolfram Research; Champaign, IL, USA) to numerically calculate pH profiles for variable *P* [6]. Then the resulting profiles were tabulated and interpolated for *P*. This interpolation function is then used in a ‘FindFit’ routine of Mathematica to extract *P* from the experimental pH profiles.

## 3. Results

**Figure 2:**
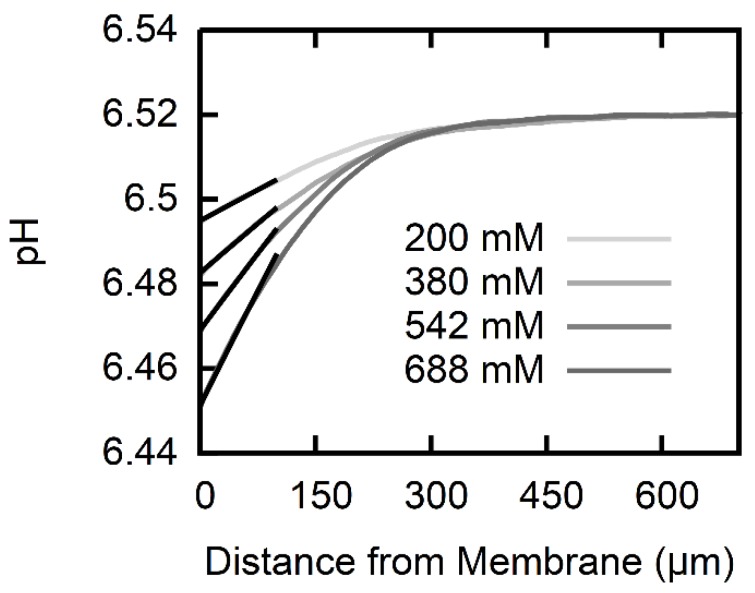
pH profiles induced by AscH transmembrane flux. The pH in the cis compartment (100 mM KCl, 0.5 mM ß-alanine, 0.3 mM KH_2_PO_4_ adjusted to pH ~6.5) acidifies within the USL, since part of the AscH molecules that arrive from the trans-compartment (100 mM KCl, 0.5 mM ß-alanine, 0.3 mM KH_2_PO_4_ adjusted to pH 4.25) deprotonate. The different concentrations of Na-l-ascorbate in the trans-compartment (see inset) range from 200 mM (light gray) to 688 mM (dark gray). Numerically calculated pH profiles (black lines) that take into account both diffusion and expenditure in protonation reactions for ascorbic acid, and the two buffering agents are fitted to the experimental data within the first 100 μm. A membrane permeability of the polar lipid extract (PLE) lipid membrane for ascorbic acid $P\;=\;1.1\;\pm \;0.1\;\times \;10^{-8}\;\mathrm{cm}/s$ is obtained.

An AscH transmembrane concentration gradient gives rise to an acid flux. In turn, AscH dissociation in the receiving USL gives rise to a pH shift (Figure 2). We optimized the pH profile size by using (i) low buffer concentrations in both compartments, (ii) large Na-l-ascorbate concentrations in the *cis* compartment, and (iii) a transmembrane pH gradient. As described in Materials and Methods, we first found δ (Equation (10)) and subsequently fitted the set of differential equations (Equations (2)–(9)) to the pH profiles to obtain *P*. Both *P* and *δ* are listed in Table 2. The USL width δ reduces with an increasing AscH gradient. This effect can be attributed to an osmotic water flux [27] that builds up due to the asymmetric addition of AscH. The resulting convection is not taken into account in the numerical calculation, but acknowledging the different δ for the different gradients yields little variance in *P* ($1.1\;\pm \;0.1\;\times \;10^{-8}\;\mathrm{cm}/s$).

**Figure 3:**
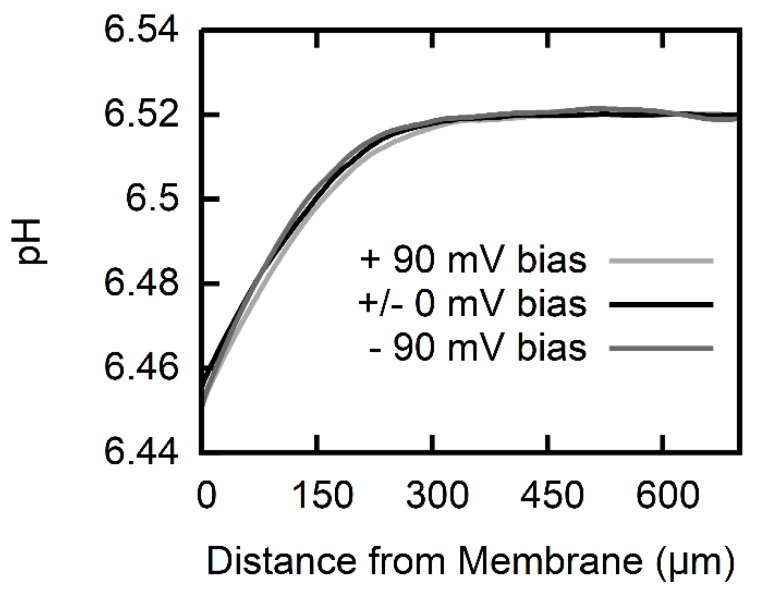
pH profiles at a constant AscH gradient (trace for 688 mM Na-l-ascorbate in Figure 2) under varying transmembrane voltage bias (black: no bias, light gray: +90 mV, dark gray: −90 mV). The pH profiles are independent of the applied transmembrane voltage within the borders of resolution which attributes the pH profiles to the permeation of a neutral species.

The pH profiles are not susceptible to the application of a transmembrane potential (Figure 3). This observation suggests that the ionic form does not permeate the membrane on a large scale. Otherwise, the ${\mathrm{Asc}}^{-}$ anions that were driven by external voltage across the membrane would have affected the deprotonation of the also permeating AscH, that is, the equilibrium AscH concentration adjacent to the receiving interface would have increased simply because the concentration of ${\mathrm{Asc}}^{-}$ increased. In turn, the transmembrane AscH concentration gradient would have diminished, resulting in a smaller flux and smaller pH profiles.

**Figure 4:**
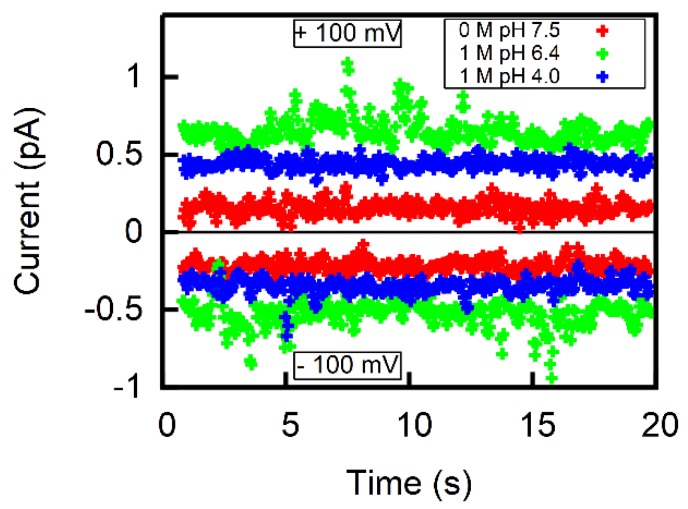
Representative current traces for ±100 mV applied to freestanding lipid bilayers of comparable sizes (diameter of aperture 395.5 µm) in the absence of Na-Asc at pH 7.5 (red) and in the presence of 1 M Na-Asc at pH 6.4 (green) and at pH 4.0 (blue). The aqueous solution contained 100 mM NaCl and 10 mM HEPES. If all conductivity is attributed to Asc^−^, one obtains an upper limit for $P^{-}$ on the order of $3\;\times \;10^{-12}\;\mathrm{cm}/s$.

Membrane conductivity measurements (Figure 4) confirmed the assumption that the ${\mathrm{Asc}}^{-}$ flux is much lower than the AscH flux. Even though the ${\mathrm{Asc}}^{-}$ concentration was raised to 1 M and pH was decreased to match acid’s pK, we did not observe an increment in current >1 pA at 100 mV of DC voltage. This translates into a specific membrane conductivity *g* < $10\;\mathrm{nS}/{\mathrm{cm}}^{2}$ for a membrane that is ~400 μm in diameter. Such *g* value is on the lower end of values reported for freestanding lipid bilayers ($10–100\;\mathrm{nS}/{\mathrm{cm}}^{2}$, [14,28]). Attributing *g* in its entirety to the permeation of ${\mathrm{Asc}}^{-}$ yields an upper limit for $P^{-}$ of $3\;\times \;10^{-12}\;\mathrm{cm}/s$ (Equation (1)). Thus, $P^{-}$ is at least four orders of magnitude smaller than *P*.

## 4. Discussion

With *P* of about $10^{-8}\;\mathrm{cm}/s$ AscH permeates fluid membranes much slower than other weak acids of comparable size—like salicylic acid [5,29] or acetic acid [6]—but is comparable to the membrane permeability of other neutral substances, for example, sorbitol [30]. The transport rate of the deprotonated species ${\mathrm{Asc}}^{-}$ is at least four orders of magnitude lower, being characterized by $P^{-}\;<3\times \;10_{-12}\;\mathrm{cm}/s$. It is thus even smaller than to the slow transport rate of the smaller chloride ions, for which $10^{-11}\;\mathrm{cm}/s$ were reported [31].

Our study is not in line with data reported by a nuclear magnetic resonance (NMR) study, where $2.6\;\times \;10^{-10}\;\mathrm{cm}/s$ and $3\;\times \;10^{-8}\;\mathrm{cm}/s$ have been deduced for ${\mathrm{Asc}}^{-}$ and AscH effluxes from fluid dipalmitoyl-lecithin (DPPC) vesicles at 52 °C [9]. The small difference between *P* and $P^{-}$ in the NMR study seems to violate membrane electrostatics: the latter imposes a penalty for placing a monovalent ion with a gyration radius *r* = 4.9 Å into the bilayer [32] of about 16.5 kcal/mol [33]. Substracting (i) 5.4 kcal/mol with which the dipole potential favors anion permeation and (ii) 2.5 kcal/mol for the image energy [34], we find an additional penalty of 8.6 kcal/mol for the anion, which translates into a drop of roughly six orders of magnitude in permeabilities as compared to the neutral species. In contrast, the NMR data favor the permeation of the neutral species by only a factor of 100.

A comparison with our data suggests that *P* has been underestimated and $P^{-}$ overestimated in the NMR study: the NMR-based AscH rate transforms into *P* $\approx 10^{-9}\;\mathrm{cm}/s$ for a fluid bilayer at room temperature, which amounts to only 1/10 of the permeability of our planar bilayers. The calculation assumes that the activation energy *E*_A_ scales with *P* [35], that is, amounts to *E*_A_ ≈ 20 kcal/mol for AscH—a value similar to that of tetraphenylborate [34] that translocates at $10^{-7}\;\mathrm{cm}/s$ [36]. The ${\mathrm{Asc}}^{-}$ rate of the NMR study is equivalent to $P^{-}\approx {\;10}^{-12}\;\mathrm{cm}/s$ for a fluid bilayer at room temperature, that is, it matches the value of the upper permeability limit of planar bilayers. The calculation assumes *E*_A_ ≈ 30 kcal/mol [34]—as has been reported for Cl^-^ that permeates at  30 kcal/mol [34]—as has been reported for Cl^-^ that permeates at $10^{-11}\;\mathrm{cm}/s$ [34].

Attempts to derive *P* and $P^{-}$ from the reaction of ${\mathrm{Asc}}^{-}$ with paramagnetic spin probes that were intercalated in oriented lipid multilayers [10] resulted in a large overestimation of the transport rate $P^{-}$. It was estimated to be $\approx 10^{-7}\;\mathrm{cm}/s$. If the calculation was correct, ${\mathrm{Asc}}^{-}$ would appear inside lipid vesicles of diameter *d* = 100 nm after time [37]

(11) $$\tau =\frac{d}{6\cdot P^{-}}=\frac{10^{-5}\;\mathrm{cm}}{6\cdot 10^{-7}\;\mathrm{cm}\;s^{-1}}=17\;s,$$

had elapsed subsequent to ${\mathrm{Asc}}^{-}$ addition to the outer solution. Yet, both a recent time resolved electron paramagnetic resonance(EPR) immersion depth study [38] and the original EPR study [10] indicate that it takes ${\mathrm{Asc}}^{-}$ 20–30 min to penetrate to a probe that is buried at a depth of 20 Å of a fluid lipid bilayer. The $P^{-}$ of $\approx 10^{-7}\;\mathrm{cm}/s$ would completely rule out the possibility of using ascorbate to monitor lipid flip-flop [39], the more so, the neutral species AscH would permeate 10^6^ times faster (see Equation (11)).

According to Overton’s rule, both *P* and $P^{-}$ correlate well with the partition coefficients *K_oct_*_/*w*_ and *K_oil_*_/*w*_ between water and octanol or olive oil, respectively [40]:

(12) $$\log P=1.154\cdot \log K_{oct/w}-2.192,$$

(13) $$\;\log P=1.132\cdot \log K_{\frac{oil}{w}}-0.679.\;$$

Inserting published *K_oct_*_/*w*_ and *K_oil_*_/*w*_ into the empirical relations Equations (12) and (13) yields overestimated *P* values (Table 3). The neglect of the acid base equilibrium is a major reason for the failure. For example, Oldendorf [11] obtains *K_oil_*_/*w*_ for carbon-radiolabeled AscH in a biphasic system of Ringer’s solution buffered to pH 7.55–7.58 and refined olive oil. Dissecting the contributions of AscH and ${\mathrm{Asc}}^{-}$ by using acid’s pK and the pH in the aqueous phase may be misleading: *P* of AscH appears in the cm/s range (see Table 3). As pointed out before, such high permeability can be excluded. The measurements leading to ${\log K}_{oct/w}$ = −2.67 by HPLC with a stationary hydrocarbon phase and a mobile solvent phase [41] may have encountered the same problem: pH is not indicated. Moreover, the range of ${\log K}_{oct/w}$ of the calibration substances was limited to 0.9–6.5 [41]. Since it does not embrace ${\log K}_{oct/w}$ for AscH, we doubt the accuracy of the reported value.

Unfortunately, computations relying on structural similarity (structure-activity-relation, SAR [42,43]) or other descriptors (Linear Solvation Energy Relationship, LSER [44,45]) include substances with erroneous experimental partition coefficients, which may bias the prediction. Nevertheless, if the scatter of the empirical relations (Equations (12) and (13)) is taken into account, the two computed ${\log K}_{oct/w}\;$values based on SAR [42,43] and the LSER value of about 0.15 [45] yield a *P* prediction that is reasonably close to our experimentally determined value.

Our *P* value allows a very rough estimation of the AscH transport capacity of the intestinal tract. The flux (*Φ*) through the intestinal barrier:

(14) $$\;\Phi =A_{\mathrm{int}}\cdot P\cdot ∆c_{\mathrm{lb}}$$

depends on its area *A_int_* = 32 m^2^ [46] and AscH’s concentration difference Δ*c_lb_* between lumen and blood. Assuming that the total luminal concentration (AscH + Asc^−^) may be represented by the gastric juice concentration of 90 µmol/L and a plasma concentration of 30 µmol/L [47] yields Δ*c_lb_* = 60 µmol/L × 10^4.17–7.4^ = 35 nmol/L. The *P* value of 10^−8^ cm/s that was obtained at room temperature corresponds to *P* ≈ 5 × 10^−8^ cm/s at 37 °C—considering an activation energy of 20 kcal/mol. The resulting *Φ* = 6·10^−13^ mol/s is likely to be an overestimation since the number neglects the significant jejunal fluid secretion. Such *Φ* is clearly insufficient as it corresponds to only about 1 µg AscH per day. The required uptake of 30–180 mg [48] per day requires facilitated transport.

## 5. Conclusions

We were able to determine the membrane permeability of PLE to ascorbic acid to be $1.1\;\pm \;0.1\;\times \;10^{-8}\;\mathrm{cm}/s$ by using scanning electrochemical microscopy. The surprisingly low membrane permeability underpins the need for facilitated transport in human physiology. Furthermore, we showed that with an upper limit of $3\;\times \;10^{-12}\;\mathrm{cm}/s$, the membrane permeability of ascorbate is negligible as compared to the membrane permeability of ascorbic acid. This clarifies a long lasting discrepancy among available literature data for *P* and partition coefficients. The extremely low membrane permeability of ${\mathrm{Asc}}^{-}$ is in line with (i) ${\mathrm{Asc}}^{-}$’s application as quencher of reactive oxygen species that is active in aqueous solutions and (ii) its use as “membrane impermeable” quencher of EPR probes.

## Figures and Tables

**Table 1:** Parameters used for the calculation of *P* from pH profiles that have been induced by the transmembrane AscH flux.

Species	Symbol	Index *i*	D_i_ (10^−10^ m²/s)	pK	Equilibrium Constant (*K_i_*)
Protons	$H^{+}$	1	93.1 [6]	14 [23]	$10^{-8}{\;\mathrm{mM}}^{2}$
Hydroxide anions	${\mathrm{OH}}^{-}$	2	52.6 [6]	14 [23]	$10^{-8}{\mathrm{mM}}^{2}$
Ascorbic acid, ascorbate	AscH, ${\mathrm{Asc}}^{-}$	8, 7	5.97 [24]	4.17 [4]	$6.76\;\times \;10^{-2}\;\mathrm{mM}$
β-alanine zwitterion and cation	B, ${\mathrm{BH}}^{+}$	3, 4	9.36 [25]	3.63 [4]	0.234 mM
Hydrogen phosphate, dihydrogen phosphate	${\mathrm{HPO}}_{4}^{2-}$, $H_{2}{\mathrm{PO}}_{4}^{-}$	5, 6	10.41 [26]	7.21 [22]	$6.16\;\times \;10^{-5}\;\mathrm{mM}$

The calculation considers the indicated species (with index *i* for nomenclature). Diffusivities *D_i_* and pK values are from sources denoted next to the value. Equilibrium constants are recalculated from the pK value with respect to the stoichiometry of the reaction.

**Table 2:** Unstirred layer width and AscH acid permeability *P* for various gradients of Na-Asc.

Na-l-Ascorbate Gradient (mM)	USL Width δ (μm)	AscH Membrane Permeability *P* (cm/s)
200	260	$1.15\;\times \;10^{-8}$
380	245	$1.03\;\times \;10^{-8}$
542	215	$1.03\;\times \;10^{-8}$
688	195	$1.19\;\times \;10^{-8}$
Average		$1.1\;\pm \;0.1\;\times \;10^{-8}$

**Table 3:** Partition coeffcients (log *K* or *K*, the respective value is underlined) available in literature. Permeabilites based on these log *K* or *K* are estimated with the correlation (see Equations (12) and (13)) of Walter and Gutknecht [40].

Year and Source	Solvents	Method	Log *K*	*K*	Log *P* (log cm/s)	*P* (cm/s)
1974 [11]	Water/olive oil	Shake-flask method and radioactively labeled AscH/${\mathrm{Asc}}^{-}$, fraction of radioactivity in both phases	−2.34	0.0046	−3.33	$4.7\;\times \;10^{-4}$
Ibidem with correction for uncharged fraction			1.04	10.96	−0.3	0.49
1990 [49]	Water/octanole	Calculated with quantitative structure-activity relation [43]	−2.0482	0.0089	−4.55	2.8 × 10^−5^
2012 [50]	Water/octanole	Calculated with quantitative structure-activity relation [42]	−2.41	0.0039	−4.97	$1.1\;\times \;10^{-5}$
2016 [41]	Water/octanole	HPLC-based assay	−2.67	0.0021	−5.27	5.3 × 10^−6^
2017 [44]	Water/octanole	Calculated with Linear Solvation Energy Relationship (LSER) approach	−2.61	0.0025	−5.2	$6.3\;\times \;10^{-6}$
2017 [44]	Water/olive oil	Calculated with LSER	−4.4	0.00004	−5.7	$2.2\;\times \;10^{-6}$
